# Metabolomic and microbiome analysis of the protective effects of Puerarin against *Salmonella Enteritidis* Infection in chicks

**DOI:** 10.1186/s12917-023-03806-x

**Published:** 2023-11-21

**Authors:** Yu Lu, Shihao Ge, Haili Zhang, Wen Lu, Xiangli Bao, Shiling Pan, Quanhai Pang

**Affiliations:** 1 College of Veterinary Medicine, Shanxi Agricultural University, Taigu, 030801 Shanxi China; 2 College of Agriculture and Bioengineering, Heze University, Heze, 274000 Shandong China; 3 College of Pharmacy, Heze University, Heze, 274000 Shandong China

**Keywords:** Puerarin, Chicks, *Salmonella Enteritidis*, Growth performance, Metabolomic

## Abstract

**Background:**

*Salmonella Enteritidis* is a zoonotic pathogen and poses a substantial risk to human health, as well as significant financial losses to the livestock and poultry industries. It is currently urgent to identify alternatives to antibiotic treatment.

**Results:**

In this study, we explored the influence of Puerarin on the immunological response, intestinal flora, serum metabolome, and growth performance of chicks infected with *Salmonella Enteritidis*. Chicks were weighed at specific time points and the average daily gain (ADG) was calculated. Serum, intestinal, and cecal content samples were collected on days 10 and 17. The results showed that 100 mg/kg of Puerarin significantly suppressed inflammation and enhanced immune function. Metabolomic analysis showed significant differences in serum metabolites after Puerarin treatment and suggested that Puerarin may regulate abnormal amino acid and lipid metabolism after *Salmonella Enteritidis* infection through the autophagic and ABC transporter pathways. In addition, Puerarin suppressed *Salmonella Enteritidis*-induced intestinal flora dysbiosis through modulation of the microbial community structures (increased Lactobacillus, Faecalibacterium, and Subdoligranulum), as demonstrated by 16S rRNA analysis.

**Conclusions:**

In conclusion, Puerarin can improve growth performance in chicks, suppress the inflammatory response in vivo, enhance immunity, and regulate lipid and amino acid metabolism and the intestinal flora.

## Background

*Salmonella* represents a major foodborne pathogen that poses a substantial risk to both public health and livestock rearing [1]. Poultry is the main target of *Salmonella Enteritidis* infection, with chicks less than one week old being especially susceptible, and acute infection can lead to the death of the chicks [2]. Infected meat and egg products can become carriers of *Salmonella Enteritidis* and infect humans through the food chain, thus endangering public health [3]. While antibiotics are frequently used therapeutically in the livestock industries, their unjustified long-term usage to prevent and treat *Salmonella* infections has resulted in significant resistance [4]. The misuse of antibiotics in poultry farming also presents a danger to public health [5, 6]. Therefore, there is a significant need to identify safe and effective drugs to replace antibiotics, thus contributing to healthy livestock and poultry farming. Plant extracts have been identified as suitable alternatives to antibiotics because of their effective antibacterial and antimicrobial effects [7].

The primary active element of Pueraria is Puerarin, a flavonoid active substance extracted from the plant [8] that has antibacterial, anti-inflammatory, antioxidant, and immune-enhancing effects [9]. The Chinese Pharmacopeia lists Pueraria species, and they are used to cure a variety of illnesses and to maintain health [10]. Studies have reported that Puerarin markedly reduces the inflammatory response by reducing the production of nuclear factor kappaB (NF-κB) and other pro-inflammatory mediators in a mouse model of colitis [11]. When pigs were infected with epidemic diarrhea virus, it was discovered that oral administration of Puerarin reduced morbidity and enhanced the intestine's anti-inflammatory and antioxidant functions, as well as strengthening the intestinal mucosal barrier and increasing the abundance of beneficial intestinal bacteria [12]. Puerarin has also been shown to be an efficient inhibitor of the inflammatory response and apoptosis induced by Mycoplasma gallisepticum, together with protecting the lungs against damage caused by Mycoplasma gallisepticum infection [13]. However, there are no reports on the effects of Puerarin on *Salmonella Enteritidis* infection and intestinal damage in poultry. In this study, we established a chick model of *Salmonella Enteritidis* infection to investigate the preventive effects of Puerarin. We also studied changes in the composition of the cecal flora using 16S rRNA analysis and detected the changes in the serum metabolome in chicks using HPLC and MS metabolomics to clarify the mechanism of action of Puerarin.

## Results

### Puerarin improved the growth performance

Table 1 includes the growth performance metrics from the two growth phases of the chicks. It can be seen that one to six days before infection with *Salmonella Enteritidis* there was no significant effect (*P* > 0.05) on either the ADG and ADFI of the chicks in each Puerarin dose group compared with the CON group. In the second stage (days7-10), chicks of S group which infected with Salmonella showed reduced growth performance with higher ADFI and F/G values and lower ADG values compared with the CON group, with significant changes in the ADG and F/G (*P* < 0.05). Compared with CON group, the ADG values in P50, P100, P200, and P400 groups were decreased; however, the F/G values in P50, P100, P200, and P400 groups were increased. In the third stage (days11-17), the S group showed lower ADG and ADFI values and higher F/G values compared with the CON group (*P* < 0.05); there were no significant differences in ADG, ADFI and F/G values between the P100 and CON groups (Table 1).

**Table 1:** Influence of Puerarin food supplementation on the growth performance of chickens infected with Salmonella Enteritidis

Items^1^	CON	S	P50	P100	P200	P400	SEM^2^	*P*-value
ADG(1-6d),g	2.06	1.98	1.96	2.06	1.91	1.95	0.64	0.246
ADFI(1-6d),g	2.26	2.37	2.35	2.31	2.24	2.27	0.14	0.651
F/G(1-6d)	1.15	1.23	1.21	1.12	1.21	1.14	0.76	0.278
ADG(7-10d),g	2.65^a^	0.33^c^	0.72^bc^	1.14^b^	1.04^b^	1.08^b^	0.791	0.001
ADFI(7-10d),g	3.80	3.82	3.60	3.45	3.37	3.52	0.202	0.407
F/G(7-10d)	1.44^d^	10.08^a^	5.12^b^	3.03^c^	3.25^c^	3.26^c^	0.301	< 0.001
ADG(11-17d),g	11.86^a^	8.26^c^	10.43^abc^	10.85^ab^	9.22^bc^	9.23^bc^	0.98	0.002
ADFI(11-17d),g	14.91^ab^	13.38^bc^	13.55^abc^	15.02^a^	14.46^abc^	13.01^c^	0.719	0.001
F/G(11-17d)	1.27^c^	1.61^a^	1.31^c^	1.34^c^	1.58^ab^	1.42^bc^	0.301	< 0.001

^1^*Abbreviations*: *CON* control group, *S Salmonella Enteritidis* infection group, *P50 Salmonella Enteritidis* infection + 50 mg/kg Puerarin group, *P100 Salmonella Enteritidis* infection + 100 mg/kg Puerarin group, *P200 Salmonella Enteritidis* infection + 200 mg/kg Puerarin group, *P400 Salmonella Enteritidis* infection + 400 mg/kg Puerarin group

^2^*SEM* Standard error of the mean (*n* = 6)

^abcd.^The superscript of different letters in the same row indicates significant difference (*P* < 0.05)

### Morphological changes in chick intestinal tissues

**Fig. 1:**
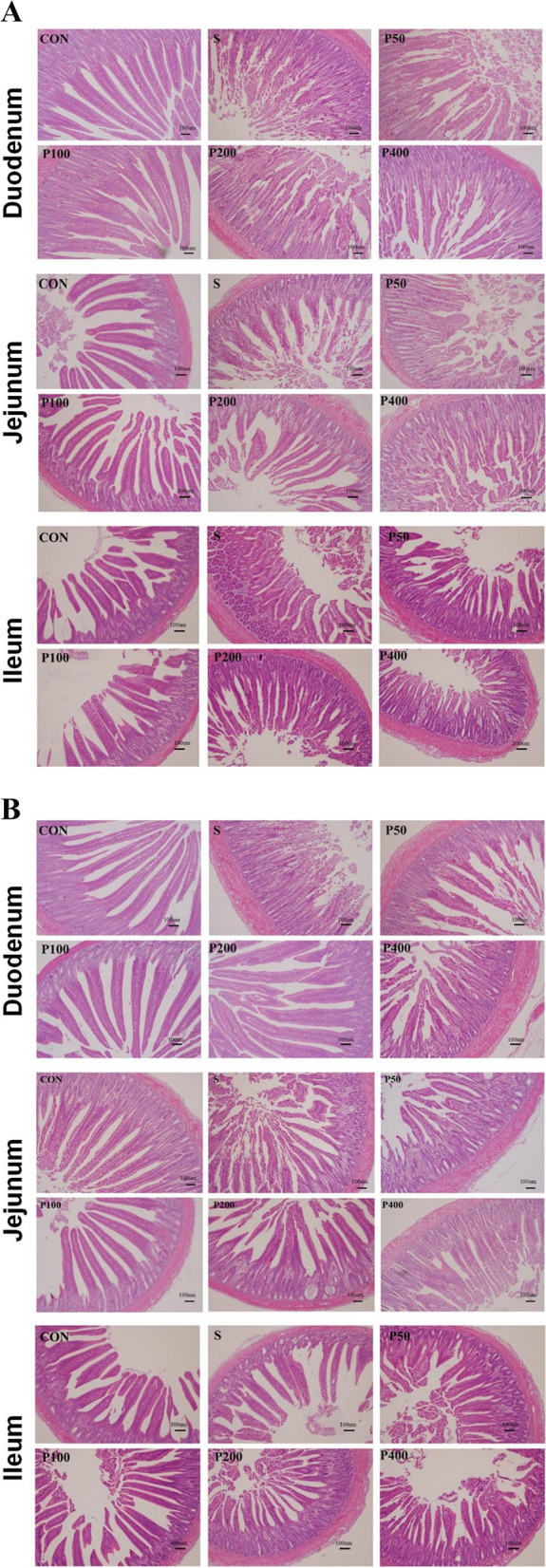
HE staining of intestinal tissue from chicks of different ages. **A** HE staining of tissue sections of duodenum, jejunum, and ileum from 10-day-old chicks. **B** HE staining of tissue sections of duodenum, jejunum, and ileum from 17-day-old chicks. Scale bar = 100 µm. CON, control group; S, the group infected with *Salmonella Enteritidis*; P50, the group infected with *Salmonella Enteritidis* + 50 mg/kg Puerarin; P100, the group infected with *Salmonella Enteritidis* + 100 mg/kg Puerarin; P200, the group infected with *Salmonella Enteritidis* + 200 mg/kg Puerarin; P400, the group infected with *Salmonella Enteritidis* + 400 mg/kg Puerarin

To further investigate the effects of Puerarin, the intestinal histomorphology of the chicks in each group was compared and evaluated. HE staining of samples collected on day 10 indicated that the intestinal epithelial villi of the duodenum, jejunum, and ileum in S group were severely damaged compared with those in group CON, with varying degrees of villus breakage, uneven arrangement of villi, and partial erosion of the mucosal layer, as well as reduced VH values and increased CD values in all intestinal segments in S group (Fig. 1). In particular, compared to the CON group, the CD values for duodenal samples in S group were significantly elevated (*P* < 0.05) while the VH:CD ratio was reduced at day 10. In contrast to the S group, the amount of damage seen in the different intestinal segments was reduced in P100 and P200 groups, and conversely, the VH value and VH:CD ratio were increased (Table 2). There were no significant differences in CD and VH:CD ratio values between the P100 and CON groups.On day 17, compared with the CON group, severe damage to the intestinal epithelial villi was observed in the S group, with significant impairment to villus uniformity and integrity; the mucosal layer was also affected to a certain extent, with increased CD and decreased VH values and VH:CD ratios (Table 2). The degree of damage to the intestinal epithelial villi was reduced in the P100 group compared with the S group, with all the Puerarin-treated groups showing reduced CD, increased VH, and increased VH:CD values. These results suggest that Puerarin can repair intestinal epithelial villus damage caused by *Salmonella Enteritidis*.

**Table 2:** Effect of puerarin on intestinal pathology of chicks challenged with *Salmonella Enteritidis*

Items	CON	S	P50	P100	P200	P400	SEM	*P*-value
Day 10								
Duodenum								
Villus height (um)	1520.75	1269.80	1656.40	1776.52	1884.08	1852.82	80.56	0.219
Crypt depth (um)	617.44^b^	799.53^ab^	800.23^ab^	796.02^ab^	965.17^a^	963.34^a^	32.28	0.01
Villus/Crypt	2.51	1.61	2.06	2.28	1.93	1.93	0.09	0.074
Jejunum								
Villus height (um)	1717.28	1441.45	1631.48	1777.28	1688.86	1564.46	82.04	0.895
Crypt depth (um)	722.85	895.00	911.51	799.80	840.09	765.29	37.13	0.684
Villus/Crypt	2.34	1.66	1.84	2.28	2.06	2.04	0.08	0.111
Ileum								
Villus height (um)	1492.53	1323.91	1400.74	1713.50	1413.87	1014.19	82.82	0.278
Crypt depth (um)	735.75	752.48	663.15	681.40	643.51	513.72	31.51	0.114
Villus/Crypt	2.03	1.75	2.16	2.62	2.17	1.86	0.10	0.163
Day 17								
Duodenum								
Villus height (um)	1853.20	1603.14	2073.07	2226.52	2417.41	2269.49	119.56	0.403
Crypt depth (um)	917.44	940.53	1066.90	846.02	1115.17	1013.34	42.46	0.482
Villus/Crypt	2.31	1.69	1.92	2.89	2.31	2.23	0.13	0.102
Jejunum								
Villus height (um)	1717.28	1506.45	1964.81	1893.95	1772.19	1847.80	87.08	0.748
Crypt depth (um)	722.85	911.67	994.85	866.47	828.43	776.96	34.59	0.259
Villus/Crypt	2.34	1.70	1.96	2.24	2.22	2.43	0.08	0.056
Ileum								
Villus height (um)	1592.53	1140.57	1350.74	1880.17	1547.20	1064.19	100.45	0.166
Crypt depth (um)	759.08	692.48	701.48	681.40	598.51	530.39	41.89	0.698
Villus/Crypt	2.25	1.67	1.98	3.82	2.61	1.87	0.26	0.194

*SEM* Standard error of the mean(*n* = 6)

^abcd^ The superscript of different letters in the same row means that the difference is significant and statistically significant. (*P* < 0.05)

### Changes in serum inflammatory factors and immunoglobulin levels

As shown in Table 3, on day 10, the IL-1β and IL-6 levels were noticeably higher in S group compared with the CON group (*P* < 0.001) while the levels of IgM were decreased, and the IgA concentrations were significantly increased (*P* < 0.001). Compared with S group, the levels of IL-1 and IL-6 first decreased and then increased as the Puerarin concentration increased, with the lowest levels seen in P100 group. In contrast, while the levels of IgM, IgG, and IgA followed the same pattern of initial increase followed by decrease, they were all highest in the P100 group. The contents of IL-1 and IL-6 were essentially similar between the P100 and CON groups while the IgM, IgG, and IgA contents in P100 group were significantly higher than those in the CON group. At day 17, the contents of IFN-γ, TNF-α, IL-6, and IL-1β were significantly higher in S group compared with those in the CON group. Conversely, the levels of IgM, IgG, and IgA were reduced, with IgM showing the most significant reductions. Compared with S group, the contents of IFN-γ, TNF-α, IL-1, and IL-6 first decreased and then increased as the Puerarin concentration rose, with the lowest values seen in P100 group. However, the contents of IgM, IgG, and IgA showed the opposite trend, first increasing and then decreasing, reaching their highest values in the P100 group. There were no significant differences in IFN-γ, TNF-α, IL-6, IL-1β, IgM, IgG, and IgA levels between the P100 and CON groups. On day 17, the concentrations of IFN-γ, TNF-α, IL-1β and IL-6 were the greatest in S group compared to the CON group. On day 10, the concentrations of IFN-γ, IL-1β and IL-6 were the greatest in S group compared to the CON group. Together, these results showed that there was an obvious inflammatory response in S group, and that 100 mg/kg Puerarin could enhance immune function and reduce the production of inflammatory factors at the same time.

**Table 3:** Effect of Puerarin on the immune function of chicks exposed to *Salmonella Enteritidis*

Items	CON	S	P50	P100	P200	P400	SEM	*P*-value
Day 10								
TNF-α(pg/ml)	67.57	62.47	67.59	66.62	66.74	64.61	0.89	*P* = 0.533
IFN-γ(pg/ml)	52.94	56.18	55.52	52.80	54.00	55.69	0.66	*P* = 0.557
IL-1β (pg/ml)	370.01^d^	539.85^a^	489.63^b^	410.07^ cd^	423.26^c^	447.18^bc^	10.17	*P* < 0.001
IL-6 (pg/ml)	20.71^d^	27.12^a^	25.30^ab^	21.20^ cd^	23.42^bcd^	23.82^bc^	0.45	*P* < 0.001
IgG (ug/ml)	1841.54^bc^	1668.19^c^	1823.99^bc^	2128.34^a^	2031.00^ab^	2019.10^ab^	35.26	*P* < 0.001
IgM (ug/ml)	607.76^ab^	523.33^c^	602.73^ab^	648.84^a^	649.14^a^	554.75^bc^	10.14	*P* < 0.001
IgA (ug/ml)	300.33^d^	317.18^c^	333.76^b^	348.08^a^	343.39^ab^	316.14^c^	3.31	*P* < 0.001
Day 17								
TNF-α(pg/ml)	62.74^c^	71.37^a^	66.31^b^	63.42^bc^	63.77^bc^	66.10^b^	0.61	*P* = 0.006
IFN-γ(pg/ml)	51.76^ cd^	60.31^a^	58.15^ab^	50.89^d^	54.80^bcd^	56.69^abc^	0.74	*P* < 0.001
IL-1β (pg/ml)	381.80^e^	531.75^a^	498.50^ab^	423.06^de^	445.12^ cd^	466.02^bc^	9.04	*P* < 0.001
IL-6 (pg/ml)	21.37^c^	29.86^a^	24.79^b^	20.68^c^	24.59^b^	23.78^b^	0.53	*P* < 0.001
IgG (ug/ml)	1800.63^ab^	1651.07^b^	1790.19^ab^	1945.55^a^	1721.28^b^	1698.65^b^	24.17	*P* = 0.003
IgM (ug/ml)	613.63^ab^	518.26^c^	590.91^ab^	657.40^a^	631.82^ab^	566.12^bc^	9.72	*P* < 0.001
IgA (ug/ml)	336.32^abc^	326.13^bc^	330.45^bc^	348.73^a^	340.19^ab^	322.39^c^	2.40	*P* = 0.009

*SEM*, Standard error of the mean(*n* = 6)

^abcd^The superscript of different letters in the same row indicates significant difference (*P* < 0.05)

### Serum metabolome analysis for untargeted metabolism

**Fig. 2:**
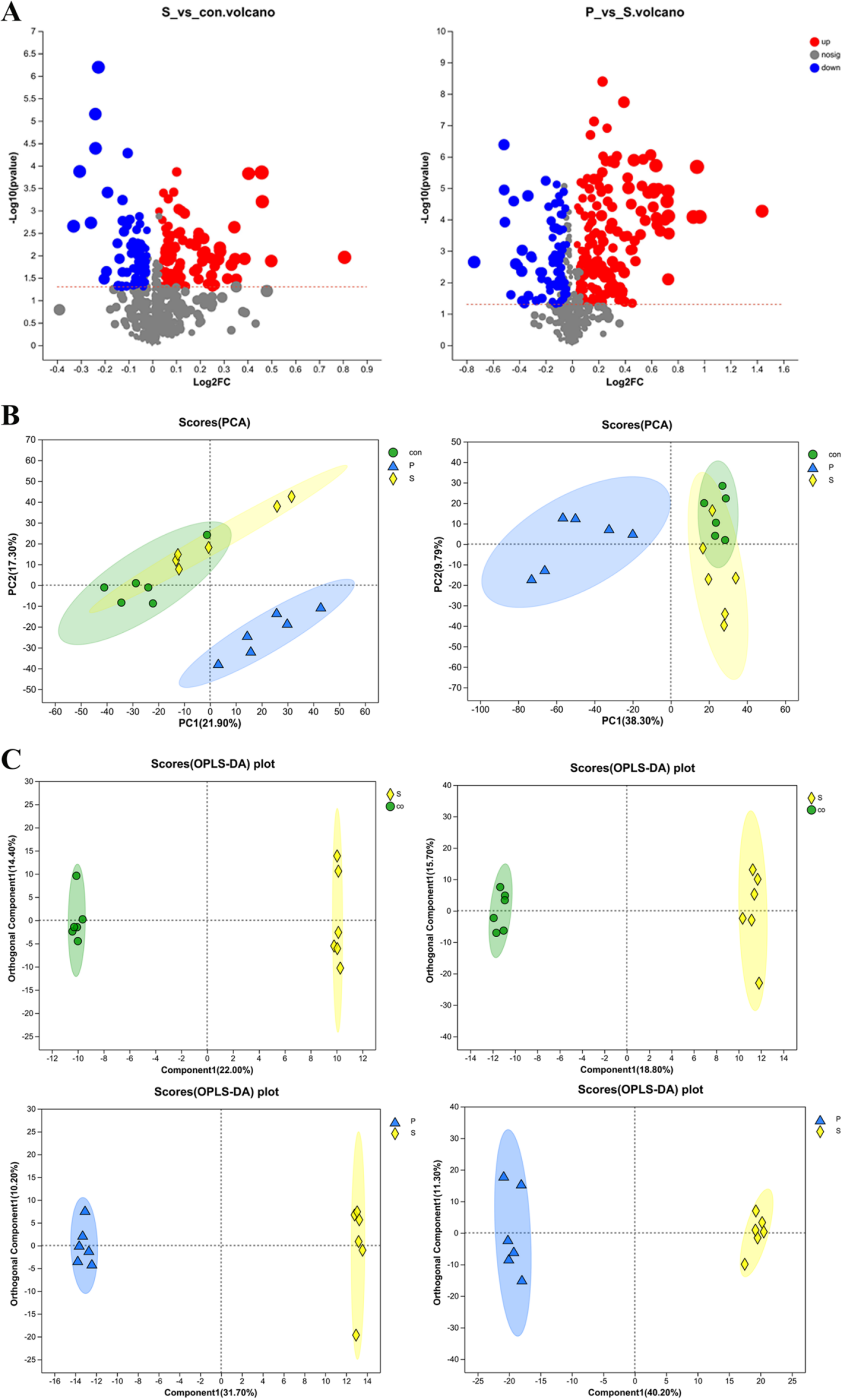
Serum metabolomic analysis between treatment groups. CON, control group; S, the group infected with *Salmonella Enteritidis*; P, the group infected with *Salmonella Enteritidis* + 100 mg/kg Puerarin at day 17. **A **Differential metabolites between the S and con groups shown in the form of volcanic plots. Differential metabolites between the S and P groups shown in the form of volcanic plots. **B **Principal component analysis (PCA) score plot for the three groups in both positive and negative ionization modes. **C **OPLS-DA score plot of metabolite profiling between the S and con groups in ESI + and ESI-; OPLS-DA score plot of metabolite profiling between the S and P groups in ESI + and ESI-

Non-targeted metabolomic analysis of sera using liquid mass spectrometry identified 767 metabolites. The overall distribution of differential metabolites between the groups was illustrated using volcano plots, where it can be seen that there were 188 differential metabolites between the CON and S groups, of which 104 were up-regulated and 84 were downregulated. A total of 258 differential metabolites were identified between the S and P groups, with 87 downregulated and 171 up-regulated (Fig. 2A). PCA was used to evaluate the separation between the different groups; this showed that the total contribution of PCA was 39.2% in the positive ion mode and 21.9% and 17.3% in PC1 and PC2, respectively. In the negative ion mode, the total contribution of PCA was 48.09%, with 38.3% and 9.79% for PC1 and PC2, respectively (Fig. 2B). Thus, while there was a trend of separation between the groups, there was also partial overlap. To further demonstrate the differences between the groups, data analysis was performed using OPLS-DA, showing significant separation between the CON and S groups while the substitution test (positive: R2Y = 1, Q2 = 0.804; negative-: R2Y = 0.999, Q2 = 0.737) indicated that the model was not overfitted, suggesting that Salmonella infection led to metabolic disturbances in the chicks. The P and S groups were markedly separated from one another in the OPLS-DA score plot, demonstrating stable metabolic differences between the two groups, and an OPLS-DA model permutation test (positive: R2 Y = 0.999, Q2 = 0.938; negative-: R2Y = 0.997, Q2 = 0.949) confirmed that the model was not over-fitted, with all Q2 values greater than 0.9, demonstrating that the model had superior prediction ability and more reliable results, suggesting that Puerarin treatment led to significant changes in the in vivo metabolism of Salmonella-infected chicks (Fig. 2C).

### KEGG pathway enrichment analysis

**Fig. 3:**
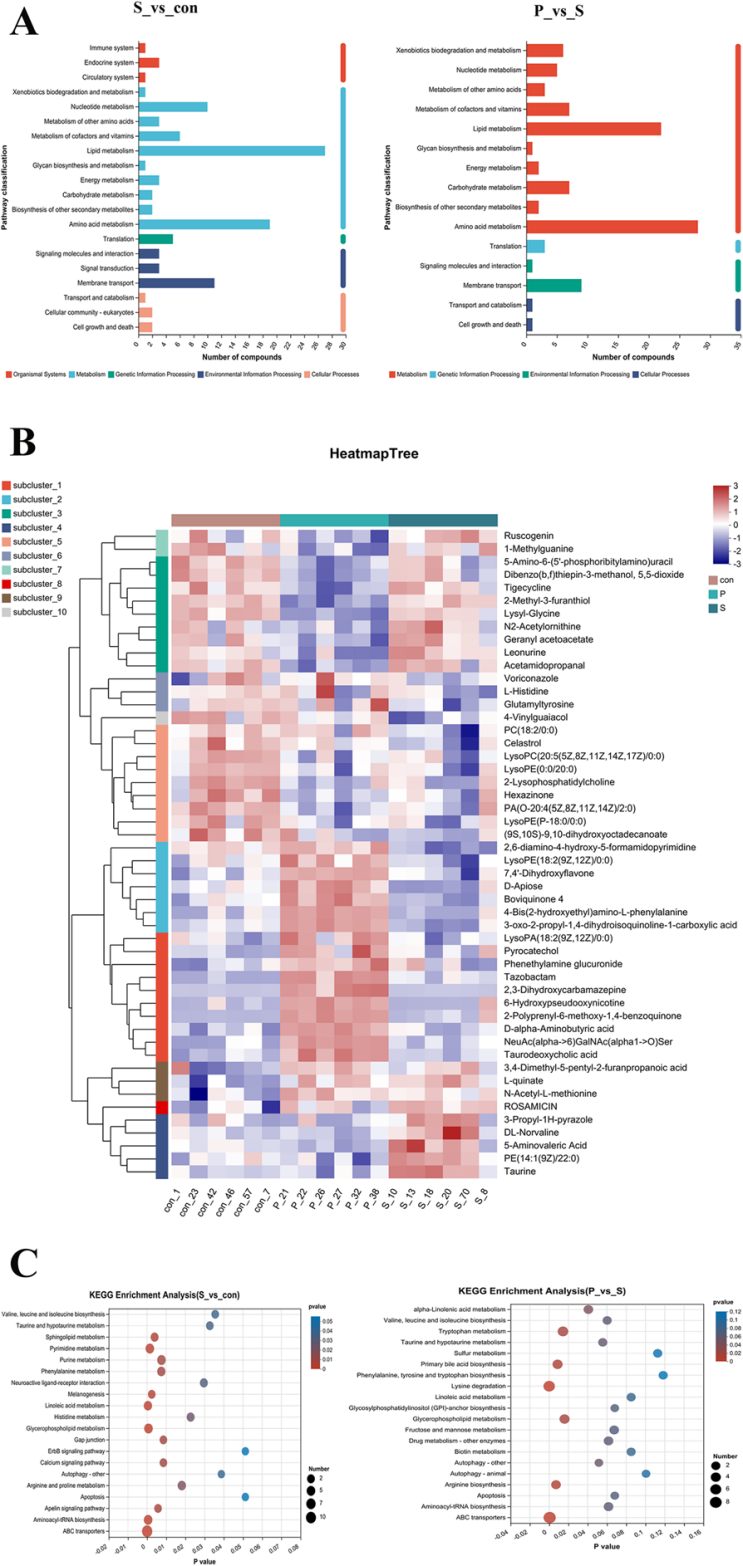
KEGG pathway enrichment analysis. **A **KEGG pathway classification between the CON and S groups; KEGG pathway classification between the S and P groups. **B **Hierarchical clustering analysis of metabolites from the CON, S, and P groups. **C **KEGG enrichment analysis shown by bubble diagram between the CON and S groups; KEGG enrichment analysis shown by bubble diagram between the S and P groups. The size of the bubble represents the number of compounds

The criteria of VIP > 1 and *P* < 0.05 were used to identify differential metabolites. Information about the pathways associated with metabolites was obtained from KEGG, and the annotation results were enriched and analyzed to identify the pathways with significant enrichment of the differential metabolites. Infection with Salmonella Enteritidis significantly impacted several pathways in comparison with the CON group. These pathways were mostly involved in "Metabolism", including: "Lipid metabolism", "Nucleotide metabolism", "Amino acid metabolism", and "Metabolism of other amino acids". In addition, several important categories were also covered, namely, "Environmental Information Processing," "Cellular Processes," "Genetic Information Processing", and "Organismal Systems" (Fig. 3A). Compared with the S group, as shown in the Fig. 3C, the P group showed significant enrichment in seven pathways, with the most common category being "Metabolism", including: "Lipid metabolism" and "Amino acid metabolism", in addition to the ABC transporter protein-associated pathway (Fig. 3C). Most of the pathways showing enrichment were associated with metabolism, suggesting that Salmonella infection affects chicken metabolism and that Puerarin alleviates the metabolic dysregulation caused by Salmonella.

Trends in the differential metabolites were evaluated using a hierarchical clustering heatmap (Fig. 3B), indicating significant differences between the CON, S, and P groups. In terms of the pathways annotated by KEGG, the most significant changes in pathways associated with lipid metabolism when compared to the CON group were observed in the S group, where three metabolites down-regulated in linoleic acid metabolism (9,10,13-TriHOME, 9(S)-HODE, 9,10-DHOME) and 12 were down-regulated in glycerophospholipid metabolism and sphingolipid metabolism (sphingosine, L-serine, sphingosine-1-phosphate) (Fig. 3C). In amino acid metabolism, a comparison between the S and CON groups showed that the S group had down-regulated levels of pathways linked to phenylalanine, tyrosine, cysteine, methionine, arginine, histidine, and proline metabolism while these effects were reversed in the P group where all these metabolic pathways were up-regulated (Fig. 3C). In addition, the ABC transporter protein, a membrane transporter protein that transports various molecules such as sugars, amino acids, and lipids involved in energy metabolism, was also significantly enriched [14]. Furthermore, the autophagy-associated pathways were significantly enriched in the S group, compared with the CON group, where there was a significant increase in the levels of autophagy-related metabolites; this trend was reversed in the P group, seen in reduced levels of autophagy-related metabolites. These findings suggest that changes in autophagy and the ABC transporter pathway indirectly affected the energy balance of the chicks, suggesting that Puerarin may regulate abnormalities in amino acid and lipid metabolism after infection with *Salmonella Enteritidis* through both autophagy and the ABC transporter pathway.

### Analysis of the microbial composition

**Fig. 4:**
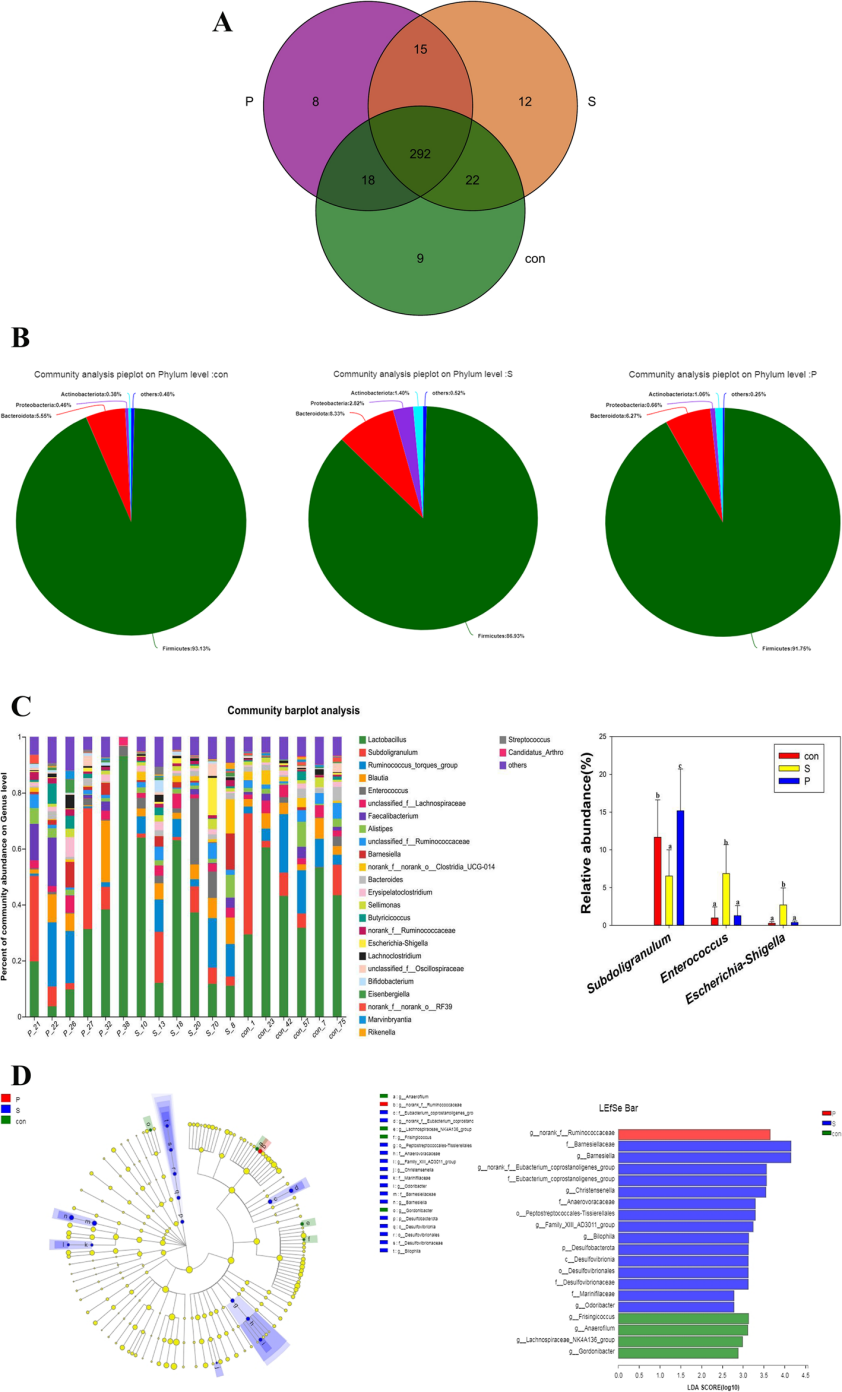
Effect of Puerarin on the microbiota of cecal contents of chicks infected with *Salmonella Enteritidis*. **A **Venn diagram of OTUs in three groups. **B **Pie chart of microbial community compositions in the three groups at the phylum level. **C** Differences in relative abundance of genera between groups and Relative abundances in the microbiota composition at the genus level. **D **Cladogram and LDA scores of gut microbiota in the three groups

The Fig. 4A showed the numbers of OTUs observed in the three groups. The OTUs in the CON group, S group and P group were 341, 341, and 333, respectively (Fig. 4A). At the phylum level, the most abundant organisms were *Firmicutes*, *Bacteroidota*, *Proteobacteria*, and *Actinobacteria*, together with a few other microorganisms (Fig. 4B). In comparison to the CON group, the abundance of *Bacteroidota*, *Proteobacteria*, and *Actinobacteria* rose while *Firmicutes* declined in the S group, indicating that Puerarin modulated the dysbiosis of the gut flora induced by *Salmonella Enteritidis* infection in chicks (Fig. 4B).

At the genus level, the relative quantities of *Subdoligranulum*, *Faecalibacterium*, and *Lactobacillus spp*. were lower in the S group compared with the CON group, whereas *Lactobacillus*, *Faecalibacterium*, and *Subdoligranulum* were found in greater relative abundance in the P group. The abundances of *Enterococcus* and *Escherichia-Shigella* were increased in the S group compared with the CON group but were decreased in the P group (Fig. 4C). Compared to the P group, the abundance of *Enterococcus* and Escherichia-Shigella have no significant change in CON group. Compared with the con group, the abundance of Lactobacillus in group S was significantly decreased. Compared with the S group, the abundance of Lactobacillus in P group was significantly increased. There were no significance between CON and P group. The CON group did not contain Salmonella. Compared with the CON group, the abundance of Salmonella in S group was significantly increased. Compared with the S group, the abundance of Salmonella in P group was significantly decreased (Fig. 4C). It has been found that *Subdoligranulum* undergoes fermentation in the intestine to create butyrate, which can protect the intestine and maintain the health of the organism [15], and it has also been found that *Subdoligranulum* has the potential to treat necrotizing colitis [16]. The abundance of *Ruminococcaceae* in the CON group is lower than that in the P group. The results of the LEfSe analysis are shown by the LDA values, indicating that, compared with the CON group, the *Eubacterium_coprostanoligenes_group, Barnesiellaceae, Christensenella, Barnesiella, norank_f__Eubacterium_coprostanoligene*s group, and *Anaerovoracaceae* were significantly more abundant in the S group, while the P group exhibited a significantly higher relative abundance of *Ruminococcaceae* than the other groups (Fig. 4D).

## Discussion

*Salmonella Enteritidis* is a major intestinal pathogen that causes severe diarrhea in poultry. It can cause sickness and death in chickens, resulting in significant financial losses as well as posing a significant risk to human health through the consumption of tainted poultry products. Pathogenic microorganisms enter the intestine of the organism by coming into contact with food or water, after which they colonize intestinal epithelial cells and subsequently invade deeper tissues, seriously harming the intestinal mucosal barrier [17]. As chicks under one week of age have immature immune systems and underdeveloped intestinal flora and mucosal barriers, they are highly susceptible to Salmonellae infection [18]. Infected chicks often show depression, reduced appetite, and white feces, often with a mushy anus, and acute infection can result in chick mortality [19]. Although antibiotics have been commonly used to prevent and treat these infections, their long-term indiscriminate use has led to significant drug resistance against *Salmonella enterica*, and we urgently need to identify and develop safe drugs to replace antibiotics in the preventive treatment of poultry diseases. Chinese herbal medicine has been found to be effective in the control of livestock and poultry diseases. Previous studies have reported that active substances from herbal preparations can be effective in preventing bacterial infections, and Puerarin is the main flavonoid active ingredient in the Chinese herb Pueraria lobata [20]. It has been reported that Puerarin significantly inhibited the inflammatory response in mice with colitis and further mitigated intestinal epithelial barrier impairment by boosting tight junction protein expression [11]. In this study, although no mortality was observed, it was found that the growth and development of the chicks were adversely affected by *Salmonella Enteritidis* infection. The addition of Puerarin to the feed was found to alleviate these problems. The findings revealed that 100 mg/kg of Puerarin substantially accelerated the chicks' daily average weight gain after infection with *Salmonella Enteritidis* and improved the growth performance of the chicks.

The primary site of nutrient digestion and absorption is the small intestine, where the small intestinal villi are crucial. The healthy development of an animal's body depends on the structural stability and proper functioning of the villi [21]. A shallower crypt suggests that the cells are maturing more quickly and have greater secretory abilities. This can be determined by the measurement of the crypt depth, while the secretory activity of the small intestine is represented by the ratio of villus height to crypt depth [22]. In this study, we found that after *Salmonella Enteritidis* infection, the villi of the small intestine appeared broken, swollen, and ulcerated, which would lead to a reduction in the intestine's capacity for digestion and absorption. The data showed that *Salmonella Enteritidis* infection decreased the VH as well as the VH:CD ratio in the small intestine, together with increasing the CD value. This would be expected to destroy the mechanical barrier provided by the small intestinal mucosa. However, the addition of 100 mg/kg of Puerarin reversed this trend, mitigating the damage to the villi and thus restoring the integrity of the small intestinal mucosa.

Chicks' immune systems are significantly activated by infection with *Salmonella Enteritidis*, resulting in an acute immunological emergency and excessive production of pro-inflammatory factors, including IL-1β, IL-6, and TNF-α [23]. Puerarin has been discovered to greatly reduce the release of pro-inflammatory mediators which, in turn, reduces the inflammatory response. IFN- γ has a key role in the activation of macrophages and the induction of major histocompatibility complex type II (MHC II) expression. One of the cytokines produced by activated macrophages is TNF, and the level of this cytokine plays a crucial role in controlling the immune response generated by the pathogen [24]. One of the interleukins in the organism that promotes the growth of immune system-related cells is IL-6. IL-1β induces the secretion of inflammatory factors from other cells, activates the proliferation and differentiation of lymphocytes, and promotes the inflammatory cell chemotaxis process [25]. In the present study, following infection with *Salmonella Enteritidis*, the levels of IL-1β, IFN-γ, TNF-α, and IL-6 rose dramatically, which is consistent with the findings of earlier research [26]. The addition of Puerarin reversed this trend, suggesting that Puerarin has an inhibitory effect on inflammation. Previous investigations have also confirmed that Puerarin has an anti-inflammatory effect, which was found to improve the anti-inflammatory function of the intestine by reducing cytokine levels in pigs infected with epidemic diarrhea [12]. It was also found that Puerarin effectively inhibited the inflammatory response induced by Mycoplasma fowleri [13]. This further explains the palliative effect of Puerarin in chicks. When chicks are infected with *Salmonella Enteritidis*, in addition to preventing the invasion of pathogens, the immune system can lessen the inflammatory response by creating antibodies. In this investigation, we discovered that on day 17, the serum IgG and IgM levels were considerably lower in the S group than in the CON group, while the serum IgA levels were lower in the S group. The levels of IgM, IgG, and IgA were noticeably elevated in the P100 group on day 17. On day 1–6, different doses of Puerarin showed no significant difference. During this time, the chicks were not infected with *Salmonella*, indicating that the different concentrations of Puerarin did not cause side effects in healthy chicks. Infection started on the sixth day of feeding and continued for three days. On day 7–17, after *Salmonella* infection, with the emergence of intestinal inflammation, immunity were weakened. During this period, the effect of 200 mg/kg and 400 mg/kg of Puerarin is not as good as that of 100 mg/kg. It was previously reported that simply raising the dosage has little effect on enhancing bioavailability and may even have negative side effects [27]. We think that for chicks with low immunity, 100 mg/kg of Puerarin has a significant effect on them, while higher concentration of Puerarin has no significant effect. These findings imply that the chick immune system was under attack and the ability to produce antibodies was suppressed by infection with *Salmonella Enteritidis*, while Puerarin can protect the immune system by triggering the release of antibodies to destroy the pathogen and suppress the inflammatory response.

Through metabolomic analysis, we demonstrated that *Salmonella Enteritidis* infection led to abnormalities in both lipid and amino acid metabolism and that Puerarin played a significant part in regulating metabolism. Puerarin has been found to modulate both glucolipid metabolism and inflammation, according to earlier studies [28]. Our findings indicated that Puerarin could increase the levels of lipid metabolism to alleviate the metabolic dysfunction caused by *Salmonella Enteritidis*. Sphingosine, L-serine, and sphingosine-1-phosphate were key factors in metabolic pathways. In our study, compared to CON group, the levels of sphingosine, L-serine, and sphingosine-1-phosphate became abnormal in S group. However, no significant differences were seen between P group and CON group in the levels of sphingosine, L-serine, and sphingosine-1-phosphate. Our findings indicated that Puerarin could alleviate the metabolic dysfunction. L-serine and sphingosine are the original substrates and sphingosine-1-phosphate is the final metabolite and is essential for controlling cellular development and survival [29]. Sphingolipid metabolites are essential in inflammation-associated signaling. Many studies have demonstrated the ability of sphingolipids to reduce inflammation [12]. The increase in these metabolites further confirms the anti-inflammatory effect of Puerarin, which is consistent with our earlier findings. Furthermore, the metabolomic analysis revealed changes in amino acid metabolism, with abnormalities in pathways associated with phenylalanine, arginine, proline, histidine, tyrosine, cysteine, and methionine metabolism; amino acids such as L-glutamic acid, L-serine, and histidine were observed to be significantly downregulated after pathogenic bacterial infection, but Puerarin reverses this trend. Glutamate, a precursor of the neurotransmitter γ-aminobutyric acid, also has a significant impact on chicken behavior and feed consumption through its concentration in the brain, while glutamate is essential for avian growth, development, and health. Glutamate is not only a significant energy source but also provides precursors for the biosynthetic activities of carbon and nitrogen [30]. In addition, changes in the autophagy and ABC transporter pathways were observed, both of which can influence energy homeostasis in chicks; a previous study reported that *Salmonella* infection can induce higher levels of autophagy in chicken jejunal tissue [31], consistent with our serum metabolome results, but Puerarin reverses this trend. Autophagy plays a significant role in the preservation of intestinal homeostasis [32]. In general, autophagy is maintained at a basic level in different tissues. It is suggested that Puerarin ameliorates abnormalities in amino acid and lipid metabolism during infection and thus alleviates the inflammatory response of the body by regulating autophagy and the ABC transporter pathway.

The intestine contains a complex microbial community that functions during digestion and metabolism, regulates nutrition synthesis, host immune system development, and control of cell division and proliferation [33]. The gut flora plays a critical role in the etiology of illnesses and acts as a biological barrier against the colonization of pathogen microorganisms [34]. The cecum of the chick is by far the most heavily populated intestinal region [35]. The present study used an analysis of the 16S rDNA of microorganisms in the cecal contents of chicks to explore the effect of Puerarin and *Salmonella Enteritidis* on the intestinal flora. The results showed that 100 mg/kg Puerarin could modify intestinal flora by controlling the composition of the microbial community structure. This was observed at the genus level where infection with *Salmonella Enteritidis* reduced the relative abundance of *Lactobacillus spp.* (*Lactobacillus*), *Faecalibacterium,* and *Subdoligranulum*. Interestingly, 100 mg/kg of Puerarin reversed this trend and increased the relative amounts of *Lactobacillus spp.* (*Lactobacillus*), *Faecalibacterium*, and *Subdoligranulum*. *Lactobacillus* is a microaerophilic Gram-positive probiotic that maintains homeostasis in the chick intestine while maintaining the natural stability of the flora [36, 37], and can also resist pathogenic bacterial infections, prevent inflammation, and regulate body metabolism [38]. In addition to being a significant butyric acid generator, *Faecalibacterium* also has anti-inflammatory properties, the ability to sustain bacterial enzyme activity, and the ability to defend the digestive tract from intestinal infections [39]. Intestinal fermentation of *Subdoligranulum* can result in the production of butyric salts, which are short-chain fatty acids that protect the intestinal tract, inhibit inflammation, and maintain the health [40]. The abundance of *Enterococcus* and *Escherichia-Shigella* was increased in the S group compared with the con group but was decreased in the P group; these represent the two main pro-inflammatory bacteria. In summary, Puerarin can mitigate the adverse effects of pathogen infection, enhance immune defense, and control intestinal morphology and the composition of the intestinal flora in chicks.

## Conclusion

In conclusion, this study constructed an in vivo model of *Salmonella Enteritidis* infection in chicks. Comprehensive analysis showed that Puerarin at a dose of 100 mg/kg was effective in protecting the chicks against the adverse effects of *Salmonella Enteritidis* infection, while improving the growth performance of the chicks, and mitigating damage to the intestinal epithelial villi, as well as inducing anti-inflammatory effects and protecting the immune system. In addition, microbial 16S rRNA and metabolome analysis showed that Puerarin could reduce both inflammation and damage induced by Salmonella Enteritidis by regulating intestinal flora and metabolism. The findings suggest that Puerarin is effective as an antibiotic substitute for reducing the risk of Salmonella Enteritidis contamination in poultry production and ensuring food safety.

## Methods

### Chemical and reagents

Puerarin (purity > 98%) was purchased from Xi’an XIAOCAO Botanical Development Co.,Ltd. (Xi’an, China). DMSO was bought from Shanghai Macklin Biochemical Co.,Ltd. (Shanghai, China). IFN-γ, TNF-α, IL-1β, and IL-6 Elisa kits were used for detecting the chick's inflammatory response. IgM, IgG and IgA Elisa kits were used for testing the ability to produce antibodies. ELISA kits were provided from Shanghai Coibo Biotechnology Co.,Ltd. (Shanghai, China). MJ-feces fecal DNA Kits were provided from Shanghai Majorbio Bio-pharm Technology Co., Ltd. (Shanghai, China).

### Animals and experimental design

All experiments were conducted in accordance with the Rules for the Administration of Laboratory Animals published by China's State Committee for Science and Technology. Two hundred and twenty one-day-old pathogen-free White Leghorn chicks were purchased from Heze Xinhang Poultry Co. Ltd. (Heze, China) and weighed. One hundred and eighty chicks, with initial body weights of 39.45 ± 2.65 g, were randomly allocated to six groups: the control group (CON), the *Salmonella Enteritidis*-infection group (S), *Salmonella Enteritidis*-infection + 50 mg/kg Puerarin group (P50), *Salmonella Enteritidis*-infection + 100 mg/kg Puerarin group(P100), *Salmonella Enteritidis*-infection + 200 mg/kg Puerarin group(P200), and the *Salmonella Enteritidis*-infection + 400 mg/kg Puerarin group (P400). Each group contained three replicates and 10 chicks. Only two chicks per replicate were used for sample collection and analysis. The CON and S groups were given a basic diet, while those chicks in P50, P100, P200, and P400 groups were fed the basal diet which supplemented with 50 mg/kg, 100 mg/kg, 200 mg/kg and 400 mg/kg body weight of Puerarin. The basal diets were developed according to the National Research Council standards (Table 4). Infection started on the sixth day of feeding and continued for three days. *Salmonella Enteritidis* was obtained from the farm, identified, and given a Gene Bank number (OP800234.1). *Salmonella Enteritidis* were thoroughly mixed in sterile water to reach the concentration of 1 × 10^8^ CFU/mL. On days 6 to 8, 1 mL (1 × 10^8^ CFU/mL) of fresh *Salmonella Enteritidis* was daily administered by mouth to the chicks through sterile pipette in S, P50, P100, P200, and P400 groups; while the CON group received an equal amount of sterile water. The chicks in each replicate were kept in separate pens and had unrestricted access to water and feed free of antibiotics. The study lasted for 17 days. The animal use protocol was reviewed and approved by Institutional Animal Care and Use Committee of Shanxi Agricultural University with approval No. SXAU-EAW-2022C.MK.004022001. The experimental procedures were performed in accordance with the Animals in Research: Reporting in vivo Experiments (ARRIVE) guidelines for reporting animal research.

**Table 4:** Composition and nutrient content of the basic diet

Item	Content
Ingredient %:	
Corn	65.70
Soybean meal	26.70
Fish meal	1.60
CaHPO4	1.28
Limestone	1.4
NaCl	0.32
Vitamin micronutrient premix^a^	3
Total	100
Calculated nutrient levels %	
Metabolizable energy (MJ/kg)	12.58
Crude protein	20
Crude fiber	6
Calcium	2.5
Total phosphorus	0.5
Lysine	0.85
Methionine	0.38

^a^The premix offered the following: vitamin A, 1500 IU; pantothenic acid, 18 mg; vitamin B1, 2.29 mg; niacin, 48.0 mg; vitamin B2, 9.0 mg; biotin, 0.15 mg; vitamin B6, 6.5 mg; vitamin D3, 2500 IU; vitamin B12, 0.02 mg; folic acid, 1.6 mg; vitamin E, 19.0 mg; Fe, 96 mg; vitamin K3, 2.8 mg; I, 0.8 mg; Zn, 86 mg; Se, 0.05 mg; Mn, 95.5 mg; Cu, 23 mg

### Sample collection

The chicks were weighed on days 1, 6, and 17 and the average daily growth (ADG) rates were calculated. The weight of feed consumed was recorded every three days to calculate the average daily feed intake (ADFI), as well as the feed to gain ratio (F/G). Samples were obtained after the chicks were weighed and sacrificed. Samples contain blood, duodenal, jejunal, ileal and cecal contents. Blood samples were collected from the wing vein of six chicks from each group on days 10 and 17, the blood was centrifuged at 4000 g for 10 min and 4 °C to obtain the serum. After the blood samples were obtained, chicks in each group were euthanized by severing jugular vessels. Chicks can lose consciousness and pain in the shortest time. The duodenal, jejunal and ileal segments were dissected and excised, rinsed slowly with PBS, and fixed with 4% paraformaldehyde. For subsequent 16S rRNA sequencing, the cecal contents were collected and stored at -80 °C for further analysis. All studies were carried out in strict accordance with relevant guidelines/regulations of the Ethics Committee on the Use and Care of Animals at Shanxi Agricultural University.

### Analysis of intestinal morphology

The fixed intestinal tissue samples were embedded in paraffin and sliced into 5-µm sections. The sections were stained with hematoxylin and eosin (HE), preserved by sealing with neutral gum, and examined under bright field using an AX70 Olympus microscope (Olympus Corporation, Tokyo, Japan). A variety of perspectives were chosen for the photographs. The villus heights (VH) and crypt depth (CD) were measured and the ratio between them (VH:CD) was calculated.

### Serum enzyme-linked immunosorbent assays

The levels of inflammatory factors (IFN-γ, IL-1β, TNF-α, and IL-6) and immunoglobulins (IgM, IgG, and IgA) were measured using ELISA kits (COIBO BIO, Shanghai, China), following the directions provided by the manufacturer.

### Serum metabolomics analysis

Metabolomic analyses were divided into three groups (*n* = 6 per group), the control group (CON), *Salmonella Enteritidis*-infection group (S), and the 100 mg/kg Puerarin treatment group (P) [13]. Serum samples obtained on day 17 were used. The metabolites were first extracted from the serum samples and the mixture was sonicated for 30 min at 40 kHz and 5 °C. After this, the samples were centrifuged for 15 min at 13 000 g and 4 °C after being kept at -20 °C for 30 min. The supernatant was then discarded.

The LC–MS analysis was performed on an ultra-high performance liquid chromatography tandem Fourier-transform mass spectrometry UHPLC-Q Exactive HF-X system (Thermo Fisher Scientific, Waltham, MA, USA). The chromatographic column was an ACQUITY UPLC HSS T3 (100 × 2.1 mm i.d., 1.8 µm; Waters, Milford, USA). The specific method of operation followed that of a previous study [41]. The source data were imported into the processing software Progenesis QI (Waters) to obtain a data matrix. Principal component analysis (PCA) and orthogonal partial least squares discriminant analysis (OPLS-DA) were performed on the Majorbio cloud platform (https://cloud.majorbio.com) using the “ropls”package in Bioconductor R software to allow comparison of metabolic differences between groups. The contribution to the classification was indicated by calculating the projection (VIP) value of each variable in the OPLS-DA model. VIP > 1.0 and *P* < 0.05 were the key criteria for screening potential metabolites. Further cluster analysis of metabolites was performed to classify pathways.

### Microbiological analysis

The total microbial genomic DNA was extracted from chick cecal contents using the MJ-feces fecal DNA Kit according to the manufacturer’s instructions. For microbiological analysis, the samples were divided into three groups (*n* = 6 per group), the control group (CON), *Salmonella Enteritidis*-infection group (S), and the 100 mg/kg Puerarin treatment group (P). Cecal contents collected on day 17 were used. The 16S rDNA high-throughput sequencing was performed by Major-bio Bio-Pharm Technology Co., Ltd. (Shanghai, China) to identify the bacterial flora in the cecal samples; the detailed steps were as described by Chen et al. [42].

### Statistical analysis

Data were analyzed with SPSS (IBM Corp., Armonk, NY, USA) and were presented as mean ± SEM. Tukey's multiple comparison test and one-way ANOVA were used to examine group differences, and SigmaPlot 12.0 was used to create histograms. Differences between groups were considered significant if *p* < 0.05.

## Data Availability

The sequencing data of *Salmonella Enteritidis* strain gene have been deposited in the NCBI Sequence repository, and the gene number is GenBank: OP800234.1. The datasets used and analyzed during the current study are available from the corresponding author on reasonable request.

## Abbreviations

CON: Control group
S: *Salmonella Enteritidis* Infection group
P50: *Salmonella Enteritidis* Infection + 50 mg/kg Puerarin group
P100: *Salmonella Enteritidis* Infection + 100 mg/kg Puerarin group
P200: *Salmonella Enteritidis* Infection + 200 mg/kg Puerarin group
P400: *Salmonella Enteritidis* Infection + 400 mg/kg Puerarin group
ADG: Average Daily Gain
ADFI: Average Daily Feed Intake
F/G: Ratio of feed to gain
ABC: ATP-binding cassette
HE: Hematoxylin-eosin
VH: Villous height
CD: Crypt depth
PCA: Principal Components Analysis
OPLS-DA: Orthogonal Partial Least Squares-Discriminant Analysis
KEGG: Kyoto Encyclopedia of Genes and Genomes
VIP: Variable important in projection
OUT: Operational Taxonomic Units
